# 4-Chloro-*N*-(2,6-dimethyl­phen­yl)benzamide

**DOI:** 10.1107/S1600536808019120

**Published:** 2008-06-28

**Authors:** B. Thimme Gowda, Miroslav Tokarčík, Jozef Kožíšek, B. P. Sowmya, Hartmut Fuess

**Affiliations:** a Department of Chemistry, Mangalore University, Mangalagangotri 574 199, Mangalore, India; b Faculty of Chemical and Food Technology, Slovak Technical University, Radlinského 9, SK-812 37 Bratislava, Slovak Republic; c Institute of Materials Science, Darmstadt University of Technology, Petersenstrasse 23, D-64287 Darmstadt, Germany

## Abstract

The conformations of the N—H and C=O bonds in the structure of the title compound (N26DMP4CBA), C_15_H_14_ClNO, are *anti* to each other, similar to that observed in *N*-phenyl­benzamide, *N*-(3,4-dimethyl­phen­yl)benzamide, *N*-(2,6-dichloro­phen­yl)benzamide and other benzanilides. There are three mol­ecules in the asymmetric unit of N26DMP4CBA. The central amide group is tilted with respect to the benzoyl ring by 45.2 (1)° in mol­ecule 1, 21.2 (2)° in mol­ecule 2 and 14.9 (2)° in mol­ecule 3. The dihedral angles between the benzoyl and aniline rings are 39.9 (1), 51.0 (1) and 86.3 (3)° in mol­ecules 1, 2 and 3, respectively. Inter­molecular N—H⋯O hydrogen bonds link the mol­ecules into infinite chains running along the [101] direction. One xylyl group is disordered over two positions; the site occupancy factors are *ca* 0.6 and 0.4.

## Related literature

For related literature, see: Gowda *et al.* (2003 ▶, 2008**a* ▶,b* ▶).
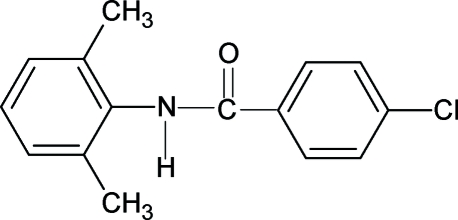


## Experimental

### 

#### Crystal data

C_15_H_14_ClNO
*M* *_r_* = 259.72
Triclinic, 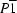

*a* = 12.2696 (3) Å
*b* = 13.6249 (4) Å
*c* = 13.7981 (4) Å
α = 91.880 (2)°
β = 113.623 (2)°
γ = 90.3676 (18)°
*V* = 2111.74 (10) Å^3^
*Z* = 6
Mo *K*α radiation
μ = 0.26 mm^−1^
*T* = 295 (2) K
0.49 × 0.22 × 0.13 mm

#### Data collection

Oxford Diffraction Xcalibur diffractometer
Absorption correction: analytical [*CrysAlis RED* (Oxford Diffraction (2007 ▶); based on Clark & Reid (1995 ▶)] *T*_min_ = 0.896, *T*_max_ = 0.973
63529 measured reflections
8072 independent reflections
3945 reflections with *I* > 2σ(*I*)
*R*_int_ = 0.042

#### Refinement

*R*[*F* ^2^ > 2σ(*F* ^2^)] = 0.046
*wR*(*F* ^2^) = 0.137
*S* = 0.89
8072 reflections
543 parameters
21 restraints
H-atom parameters constrained
Δρ_max_ = 0.21 e Å^−3^
Δρ_min_ = −0.20 e Å^−3^

### 

Data collection: *CrysAlis CCD* (Oxford Diffraction, 2007 ▶); cell refinement: *CrysAlis RED* (Oxford Diffraction, 2007 ▶); data reduction: *CrysAlis RED*; program(s) used to solve structure: *SHELXS97* (Sheldrick, 2008 ▶); program(s) used to refine structure: *SHELXL97* (Sheldrick, 2008 ▶); molecular graphics: *ORTEP-3* (Farrugia, 1997 ▶) and *DIAMOND* (Brandenburg, 2002 ▶); software used to prepare material for publication: *SHELXL97*, *PLATON* (Spek, 2003 ▶) and *WinGX* (Farrugia, 1999 ▶).

## Supplementary Material

Crystal structure: contains datablocks I, global. DOI: 10.1107/S1600536808019120/dn2356sup1.cif

Structure factors: contains datablocks I. DOI: 10.1107/S1600536808019120/dn2356Isup2.hkl

Additional supplementary materials:  crystallographic information; 3D view; checkCIF report

## Figures and Tables

**Table 1:** Hydrogen-bond geometry (Å, °)

*D*—H⋯*A*	*D*—H	H⋯*A*	*D*⋯*A*	*D*—H⋯*A*
N1—H1⋯O2	0.86	2.02	2.8585 (19)	165
N2—H2⋯O3	0.86	1.96	2.778 (2)	158
N3—H3*A*⋯O1^i^	0.86	1.99	2.814 (2)	161

Symmetry code: (i) 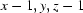.

**Fig. 1.**
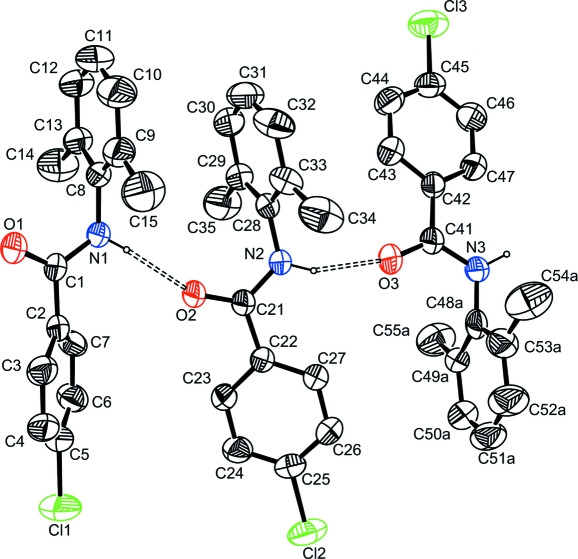
Molecular structure of the title compound showing the atom labelling scheme. Displacement ellipsoids are drawn at the 30% probability level. In molecule 3 only the A-component of the disordered xylyl ring is shown (atoms C48A to C55A). Hydrogen bonds are represented as dashed lines. H atoms not involved in hydrogen bondings have been omitted for clarity.

## supplementary crystallographic information

### Comment

In the present work, the structure of *N*-(2,6-dimethylphenyl)- 4-chlorobenzamide (N26DMP4CBA) has been determined to study the effect of substituents on the solid state geometries of benzanilides (Gowda *et al.*, 2003, 2008a,b).

The conformations of the N—H and C=O bonds in N26DMP4CBA (Fig.1) are anti to each other, similar to that observed in *N*-(phenyl)-benzamide(NPBA)(Gowda *et al.*, 2003), *N*-(3,4-dimethylphenyl)-benzamide (Gowda *et al.*, 2008a), *N*-(2,6-dichlorophenyl)-benzamide and other benzanilides (Gowda *et al.*, 2008b), with similar bond parameters. The amide group –NHCO– forms the dihedral angle of 45.2 (1)° in molecule 1, 21.2 (1)° in molecule 2, and 14.9 (2)° in molecule 3, with the benzoyl benzene ring. The dihedral angles between the benzoyl and aniline benzene rings are 39.9 (1)°, 51.0 (1)° and 86.3 (3)° in the molecule 1, 2 and 3, respectively.

The intermolecular N—H···O hydrogen bonds link the molecules into infinite chains running along the [101] direction (Table 1).

### Experimental

The title compound was prepared according to the literature method (Gowda *et al.*, 2003). The purity of the compound was checked by determining its melting point. It was characterized by recording its infrared and NMR spectra. Single crystals of the title compound were obtained from an ethanolic solution and used for X-ray diffraction studies at room temperature.

### Refinement

All H atoms attached to C atoms and N atom were fixed geometrically and treated as riding with C—H = 0.96 Å (methyl) or 0.93 Å (aromatic) and N—H = 0.86 Å with U_iso_(H) = 1.2U_eq_(Caromatic or N) and with U_iso_(H) = 1.5U_eq_(Cmethyl).

The xylyl ring of the molecule 3 revealed excessively elongated displacement ellipsoids and therefore this ring (C48 to C55) as well as the C atoms attached to it were treated as disordered with two components marked A and B. The constraint of regular hexagon was applied and the two components A and B were treated using the tools (SAME and PART) available in SHELXL97 (Sheldrick, 2008). In the first stage of refinement, the site-occupation factors were refined to be 0.561 (4) for component A (atoms C48A to C55A) and 0.439 (4) for component B (atoms C48B to C55B) then they were fixed.

### Crystal data

**Table:**

C_15_H_14_ClNO	*Z* = 6
*M**_r_* = 259.72	*F*_000_ = 816
Triclinic, *P*1	*D*_x_ = 1.225 Mg m^−^^3^
Hall symbol: -P 1	Mo *K*α radiation λ = 0.71073 Å
*a* = 12.2696 (3) Å	Cell parameters from 16102 reflections
*b* = 13.6249 (4) Å	θ = 3.2–29.3º
*c* = 13.7981 (4) Å	µ = 0.26 mm^−^^1^
α = 91.880 (2)º	*T* = 295 (2) K
β = 113.623 (2)º	Block, colourless
γ = 90.3676 (18)º	0.49 × 0.22 × 0.13 mm
*V* = 2111.74 (10) Å^3^	

### Data collection

**Table:**

Oxford Diffraction Xcalibur diffractometer	*R*_int_ = 0.042
Monochromator: graphite	θ_max_ = 25.9º
*T* = 295(2) K	θ_min_ = 5.6º
ω scans with κ offsets	*h* = −15→15
Absorption correction: analytical[CrysAlis RED (Oxford Diffraction (2007); based on Clark & Reid (1995)]	*k* = −16→16
*T*_min_ = 0.896, *T*_max_ = 0.973	*l* = −16→16
63529 measured reflections	3 standard reflections
8072 independent reflections	every 120 min
3945 reflections with *I* > 2σ(*I*)	intensity decay: none

### Refinement

**Table:**

Refinement on *F*^2^	Secondary atom site location: difference Fourier map
Least-squares matrix: full	Hydrogen site location: inferred from neighbouring sites
*R*[*F*^2^ > 2σ(*F*^2^)] = 0.046	H-atom parameters constrained
*wR*(*F*^2^) = 0.137	*w* = 1/[σ^2^(*F*_o_^2^) + (0.0808*P*)^2^] where *P* = (*F*_o_^2^ + 2*F*_c_^2^)/3
*S* = 0.89	(Δ/σ)_max_ = 0.027
8072 reflections	Δρ_max_ = 0.21 e Å^−^^3^
543 parameters	Δρ_min_ = −0.20 e Å^−^^3^
21 restraints	Extinction correction: none
Primary atom site location: structure-invariant direct methods	

### Special details

**Table:**

Geometry. All e.s.d.'s (except the e.s.d. in the dihedral angle between two l.s. planes) are estimated using the full covariance matrix. The cell e.s.d.'s are taken into account individually in the estimation of e.s.d.'s in distances, angles and torsion angles; correlations between e.s.d.'s in cell parameters are only used when they are defined by crystal symmetry. An approximate (isotropic) treatment of cell e.s.d.'s is used for estimating e.s.d.'s involving l.s. planes.
Refinement. Refinement of *F*^2^ against ALL reflections. The weighted *R*-factor *wR* and goodness of fit *S* are based on *F*^2^, conventional *R*-factors *R* are based on *F*, with *F* set to zero for negative *F*^2^. The threshold expression of *F*^2^ > σ(*F*^2^) is used only for calculating *R*-factors(gt) *etc*. and is not relevant to the choice of reflections for refinement. *R*-factors based on *F*^2^ are statistically about twice as large as those based on *F*, and *R*- factors based on ALL data will be even larger.

### Fractional atomic coordinates and isotropic or equivalent isotropic displacement parameters (Å^2^)

**Table:**

	*x*	*y*	*z*	*U*_iso_*/*U*_eq_	Occ. (<1)
C1	1.09618 (16)	0.27402 (14)	0.88857 (16)	0.0737 (5)	
C2	1.11617 (15)	0.37750 (13)	0.86787 (15)	0.0690 (5)	
C3	1.11340 (19)	0.45167 (17)	0.93676 (18)	0.0923 (6)	
H3	1.0976	0.4366	0.9952	0.111*	
C4	1.1337 (2)	0.54812 (18)	0.9205 (2)	0.1047 (7)	
H4	1.1301	0.5980	0.9667	0.126*	
C5	1.15906 (19)	0.56963 (16)	0.8358 (2)	0.0945 (7)	
C6	1.16544 (19)	0.49792 (17)	0.7682 (2)	0.0946 (6)	
H6	1.1849	0.5132	0.7118	0.114*	
C7	1.14264 (17)	0.40178 (15)	0.78394 (18)	0.0824 (6)	
H7	1.1453	0.3525	0.7367	0.099*	
Cl1	1.18407 (8)	0.69118 (5)	0.81480 (8)	0.1603 (4)	
O1	1.14255 (14)	0.24299 (11)	0.97876 (11)	0.1085 (5)	
N1	1.02664 (13)	0.21731 (10)	0.80724 (12)	0.0711 (4)	
H1	0.9923	0.2423	0.7462	0.085*	
C8	1.00731 (18)	0.11569 (14)	0.81848 (14)	0.0736 (5)	
C9	0.9194 (2)	0.08774 (18)	0.8511 (2)	0.0993 (7)	
C10	0.9064 (3)	−0.0110 (3)	0.8638 (3)	0.1357 (10)	
H10	0.8475	−0.0320	0.8852	0.163*	
C11	0.9784 (4)	−0.0784 (2)	0.8456 (3)	0.1356 (11)	
H11	0.9694	−0.1445	0.8562	0.163*	
C12	1.0624 (3)	−0.04965 (18)	0.8124 (2)	0.1176 (8)	
H12	1.1098	−0.0966	0.7990	0.141*	
C13	1.0797 (2)	0.04760 (16)	0.79793 (17)	0.0884 (6)	
C14	1.1743 (3)	0.0792 (2)	0.7626 (3)	0.1316 (9)	
H14A	1.2201	0.0235	0.7589	0.197*	
H14B	1.2254	0.1278	0.8122	0.197*	
H14C	1.1380	0.1066	0.6940	0.197*	
C15	0.8417 (3)	0.1628 (2)	0.8728 (3)	0.1510 (12)	
H15A	0.7999	0.1982	0.8099	0.227*	
H15B	0.8902	0.2079	0.9286	0.227*	
H15C	0.7853	0.1302	0.8937	0.227*	
C21	0.80003 (17)	0.33775 (13)	0.54313 (15)	0.0699 (5)	
C22	0.77906 (16)	0.43964 (13)	0.50443 (14)	0.0673 (5)	
C23	0.87450 (18)	0.50444 (15)	0.53812 (17)	0.0838 (6)	
H23	0.9490	0.4827	0.5824	0.101*	
C24	0.8632 (2)	0.59984 (15)	0.50858 (19)	0.0894 (6)	
H24	0.9293	0.6421	0.5315	0.107*	
C25	0.7536 (2)	0.63199 (14)	0.44497 (18)	0.0838 (6)	
C26	0.6571 (2)	0.57051 (16)	0.41144 (19)	0.0985 (7)	
H26	0.5824	0.5936	0.3693	0.118*	
C27	0.66980 (18)	0.47394 (14)	0.43985 (17)	0.0870 (6)	
H27	0.6038	0.4316	0.4151	0.104*	
Cl2	0.73859 (7)	0.75296 (4)	0.40748 (7)	0.1292 (3)	
O2	0.88901 (13)	0.32078 (10)	0.62217 (11)	0.1040 (5)	
N2	0.72097 (12)	0.26782 (10)	0.48919 (12)	0.0700 (4)	
H2	0.6619	0.2834	0.4326	0.084*	
C28	0.72888 (16)	0.16867 (13)	0.52006 (14)	0.0687 (5)	
C29	0.81179 (19)	0.10850 (16)	0.50619 (17)	0.0857 (6)	
C30	0.8150 (2)	0.01226 (19)	0.5342 (2)	0.1094 (8)	
H30	0.8703	−0.0293	0.5251	0.131*	
C31	0.7402 (3)	−0.0228 (2)	0.5742 (2)	0.1215 (9)	
H31	0.7445	−0.0879	0.5933	0.146*	
C32	0.6578 (3)	0.0365 (2)	0.5871 (2)	0.1237 (9)	
H32	0.6058	0.0113	0.6145	0.148*	
C33	0.6504 (2)	0.13410 (16)	0.56004 (18)	0.0921 (6)	
C34	0.5566 (3)	0.2008 (2)	0.5709 (3)	0.1433 (11)	
H34A	0.5941	0.2601	0.6097	0.215*	
H34B	0.5164	0.1678	0.6079	0.215*	
H34C	0.5000	0.2166	0.5018	0.215*	
C35	0.8955 (2)	0.1461 (2)	0.4599 (3)	0.1335 (10)	
H35A	0.9490	0.1945	0.5079	0.200*	
H35B	0.8506	0.1753	0.3935	0.200*	
H35C	0.9402	0.0926	0.4487	0.200*	
C41	0.44189 (18)	0.23143 (15)	0.21886 (17)	0.0803 (6)	
C42	0.44517 (16)	0.12663 (14)	0.18302 (16)	0.0729 (5)	
C43	0.52129 (19)	0.06379 (18)	0.25365 (19)	0.0930 (6)	
H43	0.5689	0.0876	0.3217	0.112*	
C44	0.5291 (2)	−0.03250 (19)	0.2268 (2)	0.0990 (7)	
H44	0.5801	−0.0739	0.2764	0.119*	
C45	0.4612 (2)	−0.06729 (16)	0.1265 (2)	0.0927 (7)	
C46	0.3847 (2)	−0.00681 (19)	0.0540 (2)	0.1044 (7)	
H46	0.3386	−0.0308	−0.0143	0.125*	
C47	0.37655 (19)	0.08965 (16)	0.08270 (18)	0.0908 (6)	
H47	0.3238	0.1305	0.0336	0.109*	
Cl3	0.47088 (8)	−0.18837 (5)	0.09012 (8)	0.1440 (3)	
O3	0.51840 (14)	0.26464 (11)	0.30151 (14)	0.1222 (6)	
N3	0.35416 (14)	0.28586 (12)	0.15755 (12)	0.0789 (5)	
H3A	0.2954	0.2589	0.1050	0.095*	
C48A	0.3568 (7)	0.3855 (3)	0.1777 (7)	0.080 (2)	0.56
C49A	0.4260 (6)	0.4483 (3)	0.1473 (6)	0.098 (3)	0.56
C50A	0.4161 (5)	0.5494 (3)	0.1565 (5)	0.115 (2)	0.56
H50A	0.4624	0.5914	0.1361	0.138*	0.56
C51A	0.3369 (6)	0.5877 (3)	0.1962 (5)	0.124 (3)	0.56
H51A	0.3303	0.6554	0.2024	0.149*	0.56
C52A	0.2676 (7)	0.5249 (5)	0.2267 (7)	0.139 (3)	0.56
H52A	0.2146	0.5506	0.2533	0.167*	0.56
C53A	0.2776 (8)	0.4238 (4)	0.2175 (8)	0.106 (3)	0.56
C54A	0.1967 (18)	0.3545 (10)	0.2438 (17)	0.162 (7)	0.56
H54A	0.1444	0.3920	0.2663	0.243*	0.56
H54B	0.2441	0.3132	0.2997	0.243*	0.56
H54C	0.1505	0.3144	0.1822	0.243*	0.56
C55A	0.5035 (12)	0.4070 (8)	0.0937 (10)	0.142 (4)	0.56
H55A	0.5495	0.3543	0.1339	0.213*	0.56
H55B	0.5561	0.4578	0.0899	0.213*	0.56
H55C	0.4540	0.3827	0.0235	0.213*	0.56
C48B	0.3277 (9)	0.3861 (3)	0.1819 (9)	0.077 (3)	0.44
C49B	0.3888 (8)	0.4653 (5)	0.1647 (8)	0.105 (4)	0.44
C50B	0.3633 (7)	0.5605 (4)	0.1870 (7)	0.104 (3)	0.44
H50B	0.4042	0.6135	0.1755	0.125*	0.44
C51B	0.2768 (7)	0.5765 (3)	0.2265 (6)	0.113 (3)	0.44
H51B	0.2597	0.6402	0.2415	0.135*	0.44
C52B	0.2157 (7)	0.4973 (5)	0.2438 (6)	0.113 (3)	0.44
H52B	0.1578	0.5080	0.2702	0.136*	0.44
C53B	0.2412 (8)	0.4021 (4)	0.2215 (8)	0.088 (3)	0.44
C54B	0.181 (2)	0.3162 (13)	0.244 (2)	0.134 (6)	0.44
H54D	0.2390	0.2753	0.2928	0.202*	0.44
H54E	0.1369	0.2792	0.1793	0.202*	0.44
H54F	0.1269	0.3389	0.2744	0.202*	0.44
C55B	0.4917 (12)	0.4440 (12)	0.1357 (13)	0.150 (8)	0.44
H55D	0.5192	0.5037	0.1172	0.226*	0.44
H55E	0.4669	0.3978	0.0765	0.226*	0.44
H55F	0.5549	0.4168	0.1949	0.226*	0.44

### Atomic displacement parameters (Å^2^)

**Table:**

	*U*^11^	*U*^22^	*U*^33^	*U*^12^	*U*^13^	*U*^23^
C1	0.0661 (11)	0.0725 (12)	0.0614 (13)	0.0051 (9)	0.0032 (10)	0.0065 (11)
C2	0.0613 (11)	0.0665 (12)	0.0607 (12)	0.0026 (8)	0.0054 (9)	−0.0017 (10)
C3	0.1087 (16)	0.0799 (15)	0.0766 (14)	0.0160 (12)	0.0249 (12)	0.0004 (12)
C4	0.1182 (18)	0.0728 (16)	0.0999 (19)	0.0190 (12)	0.0204 (15)	−0.0134 (13)
C5	0.0885 (15)	0.0657 (14)	0.1108 (19)	0.0000 (11)	0.0209 (14)	0.0015 (14)
C6	0.0960 (15)	0.0827 (16)	0.1026 (17)	−0.0163 (12)	0.0379 (13)	−0.0019 (14)
C7	0.0845 (13)	0.0668 (13)	0.0896 (16)	−0.0110 (10)	0.0296 (12)	−0.0121 (11)
Cl1	0.1757 (7)	0.0677 (4)	0.2189 (10)	−0.0112 (4)	0.0594 (7)	0.0131 (5)
O1	0.1175 (11)	0.0922 (10)	0.0698 (10)	−0.0058 (8)	−0.0114 (8)	0.0156 (8)
N1	0.0747 (9)	0.0635 (9)	0.0554 (9)	−0.0018 (7)	0.0051 (8)	0.0093 (7)
C8	0.0768 (12)	0.0604 (11)	0.0647 (12)	−0.0016 (10)	0.0080 (10)	0.0106 (9)
C9	0.0876 (15)	0.0877 (16)	0.1184 (18)	−0.0027 (13)	0.0357 (14)	0.0213 (13)
C10	0.127 (2)	0.107 (2)	0.176 (3)	−0.0205 (19)	0.061 (2)	0.036 (2)
C11	0.153 (3)	0.0748 (18)	0.156 (3)	−0.0189 (19)	0.037 (2)	0.0289 (17)
C12	0.139 (2)	0.0682 (16)	0.125 (2)	0.0077 (15)	0.0314 (18)	0.0068 (14)
C13	0.1003 (15)	0.0694 (14)	0.0826 (15)	0.0039 (12)	0.0230 (12)	0.0039 (11)
C14	0.156 (2)	0.110 (2)	0.159 (3)	0.0103 (17)	0.096 (2)	−0.0014 (18)
C15	0.1123 (19)	0.146 (3)	0.221 (4)	0.0118 (19)	0.092 (2)	0.027 (2)
C21	0.0672 (12)	0.0682 (12)	0.0614 (12)	−0.0022 (10)	0.0120 (10)	0.0061 (10)
C22	0.0677 (12)	0.0642 (11)	0.0611 (11)	0.0021 (9)	0.0163 (9)	0.0060 (9)
C23	0.0709 (12)	0.0728 (13)	0.0940 (15)	−0.0012 (10)	0.0177 (11)	0.0168 (11)
C24	0.0868 (15)	0.0723 (13)	0.1117 (17)	−0.0049 (11)	0.0422 (13)	0.0101 (12)
C25	0.1046 (17)	0.0592 (12)	0.0925 (15)	0.0109 (12)	0.0442 (13)	0.0084 (11)
C26	0.0864 (15)	0.0766 (15)	0.1070 (17)	0.0188 (13)	0.0113 (13)	0.0088 (12)
C27	0.0745 (13)	0.0665 (12)	0.0950 (15)	0.0021 (10)	0.0078 (11)	0.0054 (11)
Cl2	0.1546 (6)	0.0690 (4)	0.1770 (7)	0.0267 (4)	0.0777 (5)	0.0318 (4)
O2	0.0955 (10)	0.0762 (9)	0.0863 (10)	−0.0137 (7)	−0.0213 (8)	0.0215 (7)
N2	0.0625 (9)	0.0626 (9)	0.0647 (9)	0.0004 (7)	0.0044 (7)	0.0025 (7)
C28	0.0645 (11)	0.0619 (11)	0.0645 (11)	−0.0025 (9)	0.0106 (9)	−0.0037 (9)
C29	0.0812 (14)	0.0708 (14)	0.0922 (15)	0.0046 (11)	0.0215 (12)	0.0002 (11)
C30	0.1033 (18)	0.0782 (17)	0.126 (2)	0.0132 (13)	0.0247 (16)	−0.0065 (15)
C31	0.158 (3)	0.0688 (16)	0.124 (2)	−0.0065 (18)	0.041 (2)	0.0091 (15)
C32	0.159 (3)	0.093 (2)	0.133 (2)	−0.0378 (19)	0.075 (2)	−0.0056 (16)
C33	0.1031 (16)	0.0748 (14)	0.1024 (16)	−0.0176 (12)	0.0466 (14)	−0.0097 (12)
C34	0.143 (2)	0.123 (2)	0.202 (3)	−0.0182 (19)	0.112 (2)	−0.024 (2)
C35	0.1168 (19)	0.121 (2)	0.190 (3)	0.0149 (16)	0.090 (2)	−0.0027 (19)
C41	0.0716 (12)	0.0766 (14)	0.0727 (14)	−0.0032 (11)	0.0077 (11)	0.0093 (11)
C42	0.0620 (11)	0.0763 (13)	0.0718 (13)	−0.0028 (9)	0.0174 (10)	0.0110 (11)
C43	0.0879 (14)	0.0899 (16)	0.0858 (15)	0.0074 (12)	0.0180 (12)	0.0131 (13)
C44	0.0985 (16)	0.0877 (17)	0.112 (2)	0.0169 (13)	0.0414 (16)	0.0270 (15)
C45	0.0941 (16)	0.0712 (14)	0.127 (2)	0.0044 (12)	0.0588 (16)	0.0123 (15)
C46	0.1089 (17)	0.0920 (18)	0.1009 (18)	−0.0004 (14)	0.0314 (15)	−0.0131 (15)
C47	0.0871 (14)	0.0817 (15)	0.0867 (16)	0.0080 (11)	0.0168 (12)	0.0060 (12)
Cl3	0.1719 (7)	0.0809 (4)	0.1979 (8)	0.0112 (4)	0.0942 (6)	−0.0001 (4)
O3	0.1012 (11)	0.0939 (11)	0.1011 (11)	0.0008 (9)	−0.0326 (10)	−0.0029 (9)
N3	0.0770 (10)	0.0681 (10)	0.0653 (10)	−0.0031 (8)	0.0010 (8)	0.0020 (8)
C48A	0.062 (4)	0.095 (6)	0.058 (4)	−0.001 (3)	−0.002 (3)	0.008 (3)
C49A	0.065 (5)	0.064 (4)	0.135 (7)	0.003 (4)	0.010 (4)	0.004 (3)
C50A	0.106 (5)	0.088 (4)	0.126 (5)	0.012 (3)	0.021 (4)	0.011 (3)
C51A	0.161 (7)	0.081 (4)	0.102 (5)	0.030 (5)	0.025 (5)	−0.021 (4)
C52A	0.187 (9)	0.097 (6)	0.113 (5)	0.023 (6)	0.042 (5)	−0.031 (5)
C53A	0.117 (7)	0.087 (5)	0.095 (5)	−0.007 (4)	0.023 (5)	−0.006 (4)
C54A	0.24 (2)	0.134 (14)	0.136 (8)	0.050 (11)	0.096 (12)	−0.001 (10)
C55A	0.149 (7)	0.124 (6)	0.187 (11)	0.013 (5)	0.101 (8)	0.036 (6)
C48B	0.065 (5)	0.051 (5)	0.089 (6)	0.008 (3)	0.004 (4)	−0.006 (4)
C49B	0.080 (6)	0.114 (8)	0.099 (5)	−0.001 (5)	0.011 (4)	0.045 (6)
C50B	0.113 (6)	0.047 (4)	0.118 (7)	−0.011 (4)	0.011 (5)	0.013 (4)
C51B	0.129 (7)	0.080 (6)	0.082 (5)	0.005 (5)	−0.005 (4)	−0.007 (4)
C52B	0.120 (6)	0.096 (6)	0.085 (5)	−0.003 (5)	0.004 (4)	−0.017 (4)
C53B	0.099 (7)	0.063 (5)	0.066 (5)	−0.005 (5)	−0.003 (4)	−0.015 (4)
C54B	0.150 (9)	0.121 (12)	0.168 (10)	−0.033 (10)	0.103 (8)	−0.025 (11)
C55B	0.157 (15)	0.160 (15)	0.139 (10)	−0.072 (13)	0.064 (10)	0.012 (8)

### Geometric parameters (Å, °)

**Table:**

C1—O1	1.233 (2)	C33—C34	1.522 (3)
C1—N1	1.323 (2)	C34—H34A	0.9600
C1—C2	1.486 (3)	C34—H34B	0.9600
C2—C7	1.372 (3)	C34—H34C	0.9600
C2—C3	1.374 (3)	C35—H35A	0.9600
C3—C4	1.378 (3)	C35—H35B	0.9600
C3—H3	0.9300	C35—H35C	0.9600
C4—C5	1.364 (3)	C41—O3	1.220 (2)
C4—H4	0.9300	C41—N3	1.321 (2)
C5—C6	1.353 (3)	C41—C42	1.502 (3)
C5—Cl1	1.737 (2)	C42—C43	1.375 (3)
C6—C7	1.380 (3)	C42—C47	1.376 (3)
C6—H6	0.9300	C43—C44	1.366 (3)
C7—H7	0.9300	C43—H43	0.9300
N1—C8	1.427 (2)	C44—C45	1.365 (3)
N1—H1	0.8600	C44—H44	0.9300
C8—C9	1.380 (3)	C45—C46	1.368 (3)
C8—C13	1.387 (3)	C45—Cl3	1.727 (2)
C9—C10	1.379 (4)	C46—C47	1.376 (3)
C9—C15	1.506 (4)	C46—H46	0.9300
C10—C11	1.364 (4)	C47—H47	0.9300
C10—H10	0.9300	N3—C48A	1.375 (4)
C11—C12	1.345 (4)	N3—C48B	1.465 (5)
C11—H11	0.9300	N3—H3A	0.8600
C12—C13	1.374 (3)	C48A—C49A	1.3900
C12—H12	0.9300	C48A—C53A	1.3900
C13—C14	1.493 (3)	C49A—C50A	1.3900
C14—H14A	0.9600	C49A—C55A	1.520 (10)
C14—H14B	0.9600	C50A—C51A	1.3900
C14—H14C	0.9600	C50A—H50A	0.9300
C15—H15A	0.9600	C51A—C52A	1.3900
C15—H15B	0.9600	C51A—H51A	0.9300
C15—H15C	0.9600	C52A—C53A	1.3900
C21—O2	1.225 (2)	C52A—H52A	0.9300
C21—N2	1.329 (2)	C53A—C54A	1.520 (10)
C21—C22	1.490 (2)	C54A—H54A	0.9600
C22—C27	1.374 (3)	C54A—H54B	0.9600
C22—C23	1.375 (3)	C54A—H54C	0.9600
C23—C24	1.366 (3)	C55A—H55A	0.9600
C23—H23	0.9300	C55A—H55B	0.9600
C24—C25	1.363 (3)	C55A—H55C	0.9600
C24—H24	0.9300	C48B—C49B	1.3900
C25—C26	1.357 (3)	C48B—C53B	1.3900
C25—Cl2	1.732 (2)	C49B—C50B	1.3900
C26—C27	1.377 (3)	C49B—C55B	1.497 (13)
C26—H26	0.9300	C50B—C51B	1.3900
C27—H27	0.9300	C50B—H50B	0.9300
N2—C28	1.422 (2)	C51B—C52B	1.3900
N2—H2	0.8600	C51B—H51B	0.9300
C28—C33	1.375 (3)	C52B—C53B	1.3900
C28—C29	1.378 (3)	C52B—H52B	0.9300
C29—C30	1.376 (3)	C53B—C54B	1.488 (13)
C29—C35	1.505 (3)	C54B—H54D	0.9600
C30—C31	1.340 (4)	C54B—H54E	0.9600
C30—H30	0.9300	C54B—H54F	0.9600
C31—C32	1.361 (4)	C55B—H55D	0.9600
C31—H31	0.9300	C55B—H55E	0.9600
C32—C33	1.386 (3)	C55B—H55F	0.9600
C32—H32	0.9300		
			
O1—C1—N1	121.69 (18)	C33—C32—H32	119.6
O1—C1—C2	120.68 (17)	C28—C33—C32	117.9 (2)
N1—C1—C2	117.63 (16)	C28—C33—C34	120.5 (2)
C7—C2—C3	118.21 (19)	C32—C33—C34	121.6 (2)
C7—C2—C1	122.03 (18)	C33—C34—H34A	109.5
C3—C2—C1	119.7 (2)	C33—C34—H34B	109.5
C2—C3—C4	120.9 (2)	H34A—C34—H34B	109.5
C2—C3—H3	119.5	C33—C34—H34C	109.5
C4—C3—H3	119.5	H34A—C34—H34C	109.5
C5—C4—C3	119.3 (2)	H34B—C34—H34C	109.5
C5—C4—H4	120.4	C29—C35—H35A	109.5
C3—C4—H4	120.4	C29—C35—H35B	109.5
C6—C5—C4	121.1 (2)	H35A—C35—H35B	109.5
C6—C5—Cl1	119.5 (2)	C29—C35—H35C	109.5
C4—C5—Cl1	119.3 (2)	H35A—C35—H35C	109.5
C5—C6—C7	119.1 (2)	H35B—C35—H35C	109.5
C5—C6—H6	120.4	O3—C41—N3	121.64 (19)
C7—C6—H6	120.4	O3—C41—C42	120.69 (18)
C2—C7—C6	121.3 (2)	N3—C41—C42	117.67 (18)
C2—C7—H7	119.4	C43—C42—C47	117.8 (2)
C6—C7—H7	119.4	C43—C42—C41	118.59 (19)
C1—N1—C8	121.57 (15)	C47—C42—C41	123.64 (18)
C1—N1—H1	119.2	C44—C43—C42	121.9 (2)
C8—N1—H1	119.2	C44—C43—H43	119.0
C9—C8—C13	121.77 (19)	C42—C43—H43	119.0
C9—C8—N1	119.7 (2)	C45—C44—C43	119.3 (2)
C13—C8—N1	118.55 (19)	C45—C44—H44	120.4
C10—C9—C8	117.6 (3)	C43—C44—H44	120.4
C10—C9—C15	121.4 (3)	C44—C45—C46	120.4 (2)
C8—C9—C15	121.0 (2)	C44—C45—Cl3	120.2 (2)
C11—C10—C9	121.1 (3)	C46—C45—Cl3	119.4 (2)
C11—C10—H10	119.4	C45—C46—C47	119.6 (2)
C9—C10—H10	119.4	C45—C46—H46	120.2
C12—C11—C10	120.3 (3)	C47—C46—H46	120.2
C12—C11—H11	119.8	C46—C47—C42	121.0 (2)
C10—C11—H11	119.8	C46—C47—H47	119.5
C11—C12—C13	121.4 (3)	C42—C47—H47	119.5
C11—C12—H12	119.3	C41—N3—C48A	120.0 (4)
C13—C12—H12	119.3	C41—N3—C48B	126.6 (5)
C12—C13—C8	117.8 (2)	C48A—N3—C48B	15.1 (5)
C12—C13—C14	121.2 (2)	C41—N3—H3A	120.0
C8—C13—C14	121.0 (2)	C48A—N3—H3A	120.0
C13—C14—H14A	109.5	C48B—N3—H3A	111.7
C13—C14—H14B	109.5	N3—C48A—C49A	121.1 (4)
H14A—C14—H14B	109.5	N3—C48A—C53A	118.5 (4)
C13—C14—H14C	109.5	C49A—C48A—C53A	120.0
H14A—C14—H14C	109.5	C50A—C49A—C48A	120.0
H14B—C14—H14C	109.5	C50A—C49A—C55A	119.6 (5)
C9—C15—H15A	109.5	C48A—C49A—C55A	120.1 (5)
C9—C15—H15B	109.5	C49A—C50A—C51A	120.0
H15A—C15—H15B	109.5	C49A—C50A—H50A	120.0
C9—C15—H15C	109.5	C51A—C50A—H50A	120.0
H15A—C15—H15C	109.5	C50A—C51A—C52A	120.0
H15B—C15—H15C	109.5	C50A—C51A—H51A	120.0
O2—C21—N2	122.20 (17)	C52A—C51A—H51A	120.0
O2—C21—C22	119.60 (16)	C53A—C52A—C51A	120.0
N2—C21—C22	118.20 (16)	C53A—C52A—H52A	120.0
C27—C22—C23	117.79 (17)	C51A—C52A—H52A	120.0
C27—C22—C21	124.24 (17)	C52A—C53A—C48A	120.0
C23—C22—C21	117.94 (16)	C52A—C53A—C54A	120.4 (7)
C24—C23—C22	122.03 (19)	C48A—C53A—C54A	119.4 (7)
C24—C23—H23	119.0	C49B—C48B—C53B	120.0
C22—C23—H23	119.0	C49B—C48B—N3	119.8 (5)
C25—C24—C23	118.89 (19)	C53B—C48B—N3	120.2 (5)
C25—C24—H24	120.6	C48B—C49B—C50B	120.0
C23—C24—H24	120.6	C48B—C49B—C55B	117.9 (7)
C26—C25—C24	120.72 (19)	C50B—C49B—C55B	121.6 (7)
C26—C25—Cl2	120.13 (18)	C51B—C50B—C49B	120.0
C24—C25—Cl2	119.15 (17)	C51B—C50B—H50B	120.0
C25—C26—C27	119.9 (2)	C49B—C50B—H50B	120.0
C25—C26—H26	120.0	C52B—C51B—C50B	120.0
C27—C26—H26	120.0	C52B—C51B—H51B	120.0
C22—C27—C26	120.60 (19)	C50B—C51B—H51B	120.0
C22—C27—H27	119.7	C51B—C52B—C53B	120.0
C26—C27—H27	119.7	C51B—C52B—H52B	120.0
C21—N2—C28	123.50 (15)	C53B—C52B—H52B	120.0
C21—N2—H2	118.2	C52B—C53B—C48B	120.0
C28—N2—H2	118.2	C52B—C53B—C54B	120.7 (9)
C33—C28—C29	121.41 (19)	C48B—C53B—C54B	119.2 (9)
C33—C28—N2	118.86 (18)	C53B—C54B—H54D	109.4
C29—C28—N2	119.69 (18)	C53B—C54B—H54E	109.5
C30—C29—C28	118.3 (2)	H54D—C54B—H54E	109.5
C30—C29—C35	120.6 (2)	C53B—C54B—H54F	109.5
C28—C29—C35	121.2 (2)	H54D—C54B—H54F	109.5
C31—C30—C29	121.4 (3)	H54E—C54B—H54F	109.5
C31—C30—H30	119.3	C49B—C55B—H55D	109.5
C29—C30—H30	119.3	C49B—C55B—H55E	109.5
C30—C31—C32	120.3 (3)	H55D—C55B—H55E	109.5
C30—C31—H31	119.8	C49B—C55B—H55F	109.5
C32—C31—H31	119.8	H55D—C55B—H55F	109.5
C31—C32—C33	120.8 (3)	H55E—C55B—H55F	109.5
C31—C32—H32	119.6		

### Hydrogen-bond geometry (Å, °)

**Table:**

*D*—H···*A*	*D*—H	H···*A*	*D*···*A*	*D*—H···*A*
N1—H1···O2	0.86	2.02	2.8585 (19)	165
N2—H2···O3	0.86	1.96	2.778 (2)	158
N3—H3A···O1^i^	0.86	1.99	2.814 (2)	161

Symmetry codes: (i) *x*−1, *y*, *z*−1.
